# Establishing Task-Relevant MVC Protocols for Modelling Sustained Isometric Force Variability: A Manual Control Study

**DOI:** 10.3390/jfmk6040094

**Published:** 2021-11-05

**Authors:** Thomas S. Novak, Shane M. Wilson, Karl M. Newell

**Affiliations:** 1 Department of Kinesiology, University of Georgia, 330 River Rd., Athens, GA 30602, USA; shane.wilson@uga.edu (S.M.W.); kmn1@uga.edu (K.M.N.); 2 VA Rehabilitation R&D Center for Visual and Neurocognitive Rehabilitation, Atlanta VA Medical Center, 1670 Clairmont Rd., Decatur, GA 30033, USA

**Keywords:** isometric force variability, tracking error, force complexity, maximal voluntary contraction (MVC)

## Abstract

The present study examined how prevalent methods for determining maximal voluntary contraction (MVC) impact the experimentally derived functions of graded force-force variability. Thirty-two young healthy subjects performed continuous isometric force tracking (20 s trials) at 10 target percentages (5–95% MVC) normalized to a conventional discrete-point (*n* = 16), or sustained (*n* = 16) MVC calculation. Distinct rates and magnitudes of change were observed for absolute variability (standard deviation (SD), root mean squared error (RMSE)), tracking error (RMSE, constant error (CE)), and complexity (detrended fluctuation analysis (DFA)) (all *p* < 0.05) of graded force fluctuations between the MVC groups. Differential performance strategies were observed beyond ~65% MVC, with the discrete-point group minimizing their SD at force values below that of the criterion target (higher CE/RMSE). Moreover, the sustained group’s capacity to minimize SD/RMSE/CE corresponded to a more complex structure in their force fluctuations. These findings reveal that the time component of MVC estimation has a direct influence on the corrective strategies supporting near-maximal manual force control. While discrete MVC protocols predominate in the study of manual strength/endurance/precision, a 1:1 MVC-task mapping appears more to be ecologically valid if visuo-motor precision outcomes are of central importance.

## 1. Introduction

The within-subject force variability function has been investigated in both discrete and continuous isometric force paradigms. Briefly, continuous tasks require individuals to match their force output to a criterion target for a sustained duration (seconds to minutes), whereas discrete tasks involve a series of force impulses to match a parabolic target at discrete time intervals (<~500 ms). In both protocols, the amount of variability as indexed by the within-subject standard deviation (SD) increases with increments of force level, but the function tends to be linear or negatively accelerated in discrete isometric force tasks [1,2] while positively accelerated in continuous force tasks [3,4,5]. Here, we examine how the behavioral strategy of determining MVC force influences the determination of the variability function of the continuous isomeric force protocol in a way that can mediate strength/endurance/precision assessments.

The force level and the visual display conditions significantly contribute to performance variability and the dynamics of continuous tracking data in both the time and frequency domains [6,7]. At the behavioral level, few studies have provided the force–force variability function over the full range of subject’s potential force output. Initial representations of the force–force variability relation have empirically demonstrated either an exponential [4] or sigmoidal [8] function with neural drive scaling, in which the critical point for the primary increased rate of force variability change is observed at 55–65% MVC.

Christou et al. [3] examined the force variability function in the continuous isometric force task and showed a sigmoidal trend with a steep increase in variability at 65% MVC followed by a decline in rate of change at force levels above 80% MVC. In addition, they found that measuring relative force capacity provided greater validity in modeling force variability than stipulating absolute force value conditions. Christou et al. [3] interpreted their findings in relation to the size principle [9], where the prevalence of motor unit recruitment strategies occur up to ~65% MVC force, and the level of motor unit synchrony accounted for the majority of variability at higher force levels. Yao, Fuglevand, and Enoka [10] and Yao [11] expanded on this notion through modeling of motor unit recruitment/synchronization but found conflicting results. Nevertheless, there appears to be agreement on the function with recruitment strategies up to about 50/60% MVC (note: recruitment strategies can predominate out to ~85% MVC in muscles contributing to gross motor control); however, the contribution of synchrony on force variability with greater neural drive requirement has yet to be elucidated.

The majority of studies in isometric tracking have implemented a protocol where they obtain several trials in which individuals are instructed to produce 100% MVC over a range of 2–6 s [3,4,6,7,8]. The maximum force discrete point value in Newton’s (N) is obtained, and the relative MVC conditions are setup and adjusted to a % of this MVC boundary. We anticipate, however, that this technique may be more compatible for determining force capabilities in discrete isometric tasks (i.e., peak force production) given the discrete nature of the measure of force output.

In continuous force control studies, the RMSE score is typically presented to subjects after every completed trial. The RMSE accounts for variation in reference to their mean force output along with departure in relation to the target force (constant error (CE)). The goal within the task is to reduce the RMSE score as much as possible within each trial. Given that most studies take the highest absolute force value in a discrete protocol for relative %MVC calculation, it is probable that at the upper force target values, untrained individuals will be unable to maintain, in the continuous protocol, their force output with the target for a lengthy duration as a result of the influence of fatigue. This would be realized by the progressive increase in CE (that is rarely reported) at the higher force levels, particularly for the discrete MVC determination condition.

Fatigue has been operationalized as a stressor-induced (i.e., exercise, sleep deprivation, anxiety, etc.) reduction in muscle force capacity [12]. The onset of force decay (inability to maintain target force) occurs at a much faster rate than the amplification of EMG characteristics attributed to peripheral fatigue (i.e., decreased mean motor unit firing rate/increased synchrony) [10]. It is hypothesized that while motor unit synchronization may impact force steadiness, the contributions of fatigue influencing force decay are central in origin (note: the mechanisms of fatigue are contraction-intensity-dependent). Although there are conflicting formulations of the contributing mechanisms to fatigue, their relation to force variability remains ambiguous without an understanding of how the interaction between task and individual constraints influences the organizational strategies that individuals use to reduce their performance score (RMSE) across their respective force range.

The purpose of this study was to examine how the method for MVC calculation impacts the subsequent function of force–force variability based on behavioral strategies utilized by individuals to reduce variability in a continuous tracking task. We tested the proposition that mapping MVC tests to the same time duration as the relative force variability condition trials will control, to a significant degree, for the contributing factors of force decay (i.e., fatigue). The hypotheses tested were:

(1) The traditional single time peak point approach to determining MVC testing will result in strategic minimization of the magnitude of variability in relation to a mean output (SD term in RMSE calculation) at relatively high force levels. Conversely, an averaged MVC protocol matched to the trial duration will elicit strategic minimization of force variability in relation to both the mean force output and the target force (control of SD and CE).
(2) The adapted time matching protocol of MVC determination will result in reduced force variability in terms of individual SD, CE and RMSE, respectively. Consequently, the adapted MVC test will present a stronger correlation between individual SD and CE scores.
(3) There will be a qualitative difference in the time-dependent structure of individual force output, with the averaged MVC protocol demonstrating greater complexity in tracking fluctuations at higher force levels compared to the traditional discrete MVC approach.

## 2. Methods

Thirty-two (16 subjects 6 s MVC protocol, 16 subjects 20 s MVC protocol) self-reported right-handed subjects (age: 23 ± 4 years) from the university population participated in this study. The subjects were not trained in fine manual tasks, were not competitive weightlifters, and had no previous history of neurological disorder. Written informed consent was obtained from all participants in congruence with the IRB approval from The University of Georgia.

Procedures. A randomized block design was used to assign subjects to either a discrete peak MVC group (8 male, 8 female) or a trial averaged MVC group (8 male, 8 female). The experimental procedures were common across groups except for the determination of MVC. Subjects were given approximately 5 min of familiarization with the experimental apparatus and allowed to practice using the distal phalanx of the index finger protocol before testing. Participants were instructed to apply force on an entran ELFS-B3 load sensor (Entran Sensors and Electronics, Fairfield, NJ, USA) via flexion of the index finger of their dominant hand. Analog output from the force transducer was amplified (Coulbourn Instruments, Holliston, MA, USA) at an excitation voltage of 10 V and gain of 100. A 16-bit analog-to-digital (A/D) converter sampled the force output at 120 Hz. The A/D converter could detect incremental changes in force output as low as 0.0016 N. The non-dominant hand was to rest in a homologous position while testing commenced.

Upon familiarization, we performed the following MVC calculation procedures: subjects in the discrete-point MVC group produced their maximum force over 3 trials (6 s duration per trial), and the highest instantaneous force they produced was used as their MVC estimate. Subjects in the sustained MVC group produced their maximum force for 20 s in three consecutive trials. Mean force was then calculated for each trial, and the trial with the highest mean force value was used as their MVC estimate. The estimate of each subject’s MVC was then used to determine subsequent relative %MVC force conditions.

Force levels (5–95% MVC in increments of 10%) were randomized for each subject. Three consecutive trials were performed at each force condition before participants moved to the next experimental condition. Participants were instructed to produce force on the load cell so that a yellow feedback line matched a red target line in the middle of the screen. After completion of a trial, participants received knowledge of results (KR) of root mean square error (RMSE) relative to the force output.

To maintain consistency with previous studies examining graded force variability, the duration of each trial was set at 20 s [3,4,8]. In order to reduce any transient effects of fatigue between trials, subjects were provided with as much time as they needed to recover between trials that ranged from 30–60 s. Feedback of the force output was given to subjects through a 20 in (51 cm) HP monitor with a resolution of 1920 × 1080 pixels. Based on previous work from our laboratory, the force trace on the screen was set at a pixel-to-Newton ratio of 64 *p*/N.

Data Analysis. The first 3 and last 2 s of each trial were removed from the data in order to take out any major transient behavior from the tracking task. The data analysis was performed via Matlab 8.1 (Mathworks Inc., Natick, MA, USA).

Magnitude of Variability. Tracking performance as a function of force level was assessed by root mean square error (RMSE), standard deviation (SD), and constant error (CE) of the force output within each trial.

Structure of Variability. The time-dependent force structure as a function of force level was assessed by detrended fluctuation analysis (DFA) [13]. DFA integrates raw time series data and divides this integrated series into boxes with equal window length, and within each box least squares fit line is applied. In order to detrend the data, each linear fit is subtracted for each window. The root mean square (RMS) variability is calculated within each window and averaged across windows of the same size. These calculations are repeated at a range of window sizes, in which a regression slope (α) of the log-log relation between RMS variability and window size (time scale) captures the degree of self-similarity in a signal. DFA α values correspond to the degree of deterministic structure embedded in the force signal (white noise: α = 0.5, pink noise: α = 1.0, brown noise: α = 1.5). This analysis technique was utilized given the possibility that higher forces increase the probability of the signal being non-stationary.

Inferential Statistics. A mixed ANOVA with MVC as the between-subject factor (6 s and 20 s MVC groups) and force condition as the within subject factor (5–95% MVC in 10% increments) was applied on each dependent variable of interest to determine the effect of MVC protocol on the magnitude and structure of force variability as a function of force level.

All statistical analyses were significant when the probability of making a type 1 error was less than 5% (*p* < 0.05). If the assumption of sphericity using Mauchly’s test was violated, a Huynh–Feldt correction was used to adjust the statistical degrees of freedom. Only those main effects and interactions that were significant (*p* < 0.05) are reported. Analyses were performed using IBM SPSS software.

Model. Determination of model fit was performed through utilization of the *fit* function in MatLab 8.1 (Mathworks Inc.). The averaged RMSE, SD, CE, and DFA values were analyzed using *fit* (analysis of 1, 2, 3, and 4th order polynomial models) function in which determination of the best model fit was ultimately predicated on the highest R^2^ value.

Correlations. A Pearson’s product-moment correlation determined the relation of performance strategy (RMSE, SD, and CE) to MVC protocol and force level.

## 3. Results

**Figure 1:**
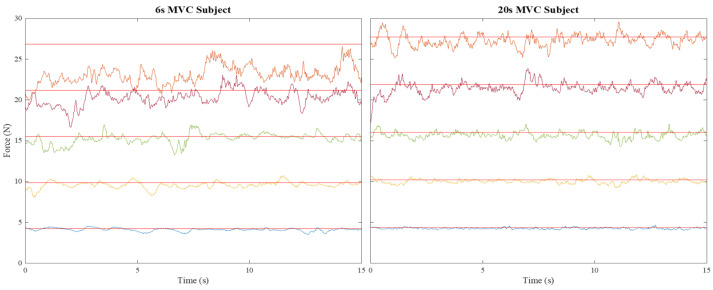
Illustrations of raw force data and corresponding targets at 15%, 35%, 55%, 75%, and 95% MVC in ascending order.

Figure 1 provides illustration of raw force data produced by two subjects in the 6 s MVC group and 20 s MVC group, respectively. The MVC trials produced the following force levels: 6 s MVC group: Mean 31.6 N, between-subject SD 14.4 N; 20 s MVC group: Mean 28.9 N, between-subject SD 7.1 N.

**Figure 2:**
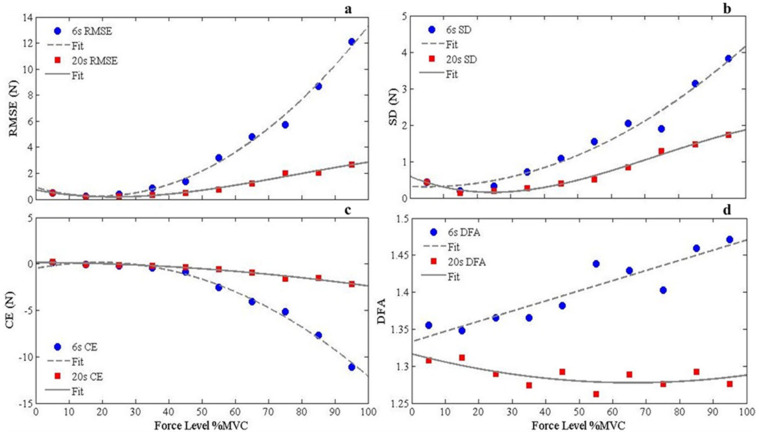
Fitted functions on averaged (**a**) RMSE, (**b**) SD, (**c**) CE, and (**d**) DFA as a function of MVC group and force level.

Figure 2 shows the average RMSE, SD, CE, and DFA values for each MVC protocol group as a function of force level along with their respective fitted functions. The respective best-fitted functions for RMSE, SD, CE, and DFA as a function of relative force output are presented in Table 1. As predicted, there were distinct differences between MVC groups in terms of RMSE, SD, and CE of absolute magnitude of performance error. Additionally, RMSE and SD showed different force variability functions between the 6 s and 20 s MVC groups, with the 6 s group producing a quadratic trend and the 20 s group demonstrating a sigmoidal trend for both respective performance outcome measures.

RMSE: There were significant main effects of MVC Group: F(1,30) = 11.94, *p* < 0.05, and Force Level: F(2.17, 65.18) = 23.34, *p* < 0.001, together with a Group x Force Level interaction: F(2.17, 65.18) = 9.80, *p* < 0.001. Post hoc analysis revealed that RMSE was significantly different between protocol groups at the 25–95% MVC conditions. Differences in within-group RMSE across force levels are shown in Table 2. Overall, there were significant difference in RMSE across force conditions for the 6 s MVC group, but not the 20 s MVC group.

SD: There were significant main effects of Group: F(1, 30) = 7.62, *p* < 0.05, Force Level: F(3.49, 104.63) = 21.05, *p* < 0.001, and a Group x Force Level interaction: (F(3.49, 104.63) = 3.28, *p* < 0.05. Post hoc analysis revealed that SD values were significantly different between force protocol groups at the 25–65%, and 95% MVC conditions. Differences in within-group SD across force levels are shown in Table 3. Overall, there were significant differences in SD across force conditions for the 6 s MVC group, but not the 20 s MVC group.

CE: There were significant main effects for Group: F(1,30) = 10.80, *p* < 0.05, and Force Level: F(2.13, 63.97) = 20.44, *p* < 0.001, with a Group x Force Level interaction: F(2.13, 63.97) = 8.98, *p* < 0.001. Post hoc analysis revealed that CE values significantly differed between force protocol groups at 35%, 45%, 65–95% MVC conditions. Differences in within-group CE across force levels are shown in Table 4. Overall, there were significant differences in CE across force conditions for the 6 s MVC group, while the 20 s MVC group showed significance differences in CE between the 15 and 35 %MVC conditions.

DFA: There was a significant main effect for Group: F(1,30) = 15.91, *p* < 0.001, and a Group x Force Level interaction: F(4.72, 141.49) = 3.38, *p* < 0.05. Post hoc analysis revealed significant differences between the force protocol groups at 25–95% MVC conditions. Differences in within-group DFA across force levels are shown in Table 5. Overall, there were significant differences in DFA across several force conditions for the 6 s MVC group but not the 20 s MVC group.

**Figure 3:**
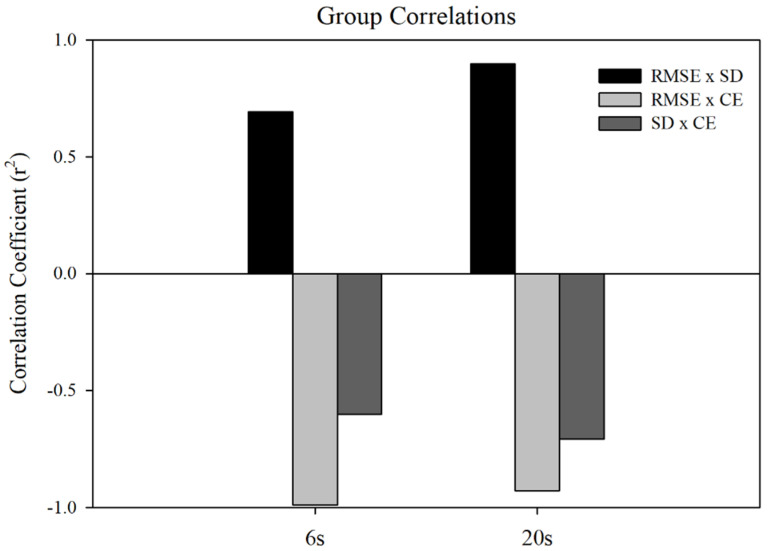
Correlations of the RMSE, SD, and CE within MVC groups.

Correlations: Figure 3 shows the correlations between RMSE and SD, RMSE and CE, and SD and CE, as a function of MVC group. The correlations were run over all Ss within each group and included all individual trials at each force level.

Both MVC protocol groups showed significant positive correlations between RMSE and SD (6 s): r^2^= 0.69, *n* = 160, *p* < 0.001, (20 s): r^2^ = 0.90, *n* = 160, and *p* < 0.001, along with significant negative correlations between RMSE and CE (6 s): r^2^ = −0.99, *n* = 160, *p* < 0.001, (20 s): r^2^ = −0.93, and SD and CE (6 s): r^2^ = −0.60, *n* = 160, *p* < 0.001, (20 s): r^2^ = −0.71, *n* = 160, *p* < 0.001, respectively. In the 6 s protocol, individuals presented a near 1:1 relation between RMSE and CE value, whereas the 20 s protocol saw a reduction in this relation along with a higher relation between SD and CE. This indicates that subjects in the 20 s MVC protocol manipulated the magnitude of mean variability and variability in relation to the target to a higher degree than the 6 s group.

## 4. Discussion

The main purpose of this study was to examine the differences in the magnitude and time-dependent structure of force variability over the potential force range when traditional MVC testing procedures were compared with a novel MVC criterion approach. Specifically, we were interested in determining whether differential mapping of MVC-experimental trial time provides insight into the disparity between the force variability functions illustrated in the contemporary literature [3,4,8]. Our results support the hypothesis that the temporal mapping and calculation of maximal force capacity to experimental conditions directly influence the behavioral strategy in reduction of performance error, and that these distinct strategies uniquely relate to the prevailing force variability functions. Additionally, the findings showed that MVC-task mapping has a direct influence on the time-dependent contribution of interacting neuro-behavioral processes in isometric tracking across the functional force range. Collectively, the results address several considerations on the modeling of isometric force tracking across the functional force range, while providing a complementary approach to compatibly account for neuro-physiologically relevant contributions in continuous force gradation.

Force Variability Functions. The general findings in continuous isometric tracking literature show that the rate of variability magnitude (SD) either continually increases towards maximal force capacity [4] or increases through the median range and decreases to asymptote beyond 70% MVC [3]. While the traditional dispersion measures of SD account for the collective magnitude of fluctuations within a relative force condition, they do not account for the accuracy (CE) as it relates to the criterion force value. Thus, RSME and SD alone do not provide insight into whether individuals can actually maintain the specified force value, thereby influencing strategic modulation of force output in order to reduce the performance feedback value generally specified by RMSE. 

A number of studies presenting the exponential force variability function have provided supplementary results on the relation between the mean relative force output (%MVC) as it relates to the target relative force [14,15], with a general finding that the mean force output is 10–15% lower than the desired force at near maximal conditions. This implies that traditional MVC-task mapping appears to be inappropriate in determining the relative force capacity of individuals for the duration of the task; however, to our knowledge there have been no experimental data as to how this constant error shift relates to the force variability function in terms of SD. Analysis of constant error (CE) can directly determine the direction and magnitude of force deviation from the target, while root mean square error (RMSE) determines the degree of fluctuation around the mean force output value along with this dispersion as it relates to the target force.

Our findings corroborate the assumption that individuals in the traditional discrete point peak MVC protocol cannot maintain the specified force for the duration of the trial given the significant negative CE and positive RMSE increase beyond ~55% MVC. Furthermore, it is apparent that this MVC group strategically selected a mean force output lower than the criterion value while modulating the degree of fluctuation around this output given a significantly lower SD value compared to their CE and RMSE scores. Conversely, the 1:1 MVC-task mapping group exhibited statistically comparable SD, CE, and RMSE values across the functional force range, thereby implying that they successfully met the criterion force value and modulated their force dispersion accordingly.

The findings show that the behavior strategy based on force output capacity has a direct influence on the force variability function. Consequently, we have established a basis for the disparity in the force variability literature, and the averaged MVC-mapping results are in agreement with the findings presented by Christou et al. (2002), who implemented a traditional MVC testing approach, in addition to not providing feedback about task performance between trials. While their subsequent analysis may have removed the transient force capacity limitations (i.e., fatigue, selection of lower force value), such an experimental design presents several issues. Firstly, such a discrete window of force output provides a limited view on the collective performance dispersion throughout the duration of the task. Secondly, the removal of performance feedback in conjunction with force output analysis in a discrete timeframe makes it impossible to assess whether their results would differ in both magnitude and function of variability as a consequence of behavioral modification. In fact, while they presented a sigmoidal force variability function, the standard deviation values were considerably greater than our averaged MVC-mapping results, with values more comparable to the traditional MVC testing group.

Previous studies have attempted to determine how changes in neurophysiological organization elicit modification in behavioral outcome with little emphasis on neuro-behavioral strategy itself. The most relevant attempts to understand how behavioral modification influences force gradation found that volitional excitatory drive to the moto-neuron pool is a successful means to counteract any detrimental changes in rate coding and synchronization strategies [16,17]. Regardless of the experimental question, continuous force production at all relative neural-drive requirements is sub-maximal compared to discrete force gradation, and the determination of force capacity should be manipulated accordingly. In fact, there is evidence that the sub-maximal maximum voluntary isometric contraction (MVIC) normalization techniques provide more validity to capturing neurophysiological outcomes when performing continuous force contractions [18,19]. Given the apparent differences in behavioral strategy with MVC-task mapping, we provide an experimental framework to the force variability function while providing an avenue for investigating the mechanistic connection of force gradation strategy when individuals have the capacity to maintain performance in the criterion task.

Force Structure. The majority of isometric force tracking studies examining the force variability function have utilized various entropy measures (approximate entropy, sample entropy, and multi-scale entropy) to determine how relative force requirement influences the structural complexity of the force signal [4,14,20]. There is a level of ambiguity to the force entropy function at low force levels; however, there is a consistent finding that high force requirements exhibit highly deterministic time-dependent variations in the signal as a consequence of the reduced functional degrees of freedom capable of modulating near maximal force control. Entropy measures are appropriate under the assumptions that the signal of interest is stationary. Given our results of CE, and RMSE, utilization of non-linear time-dependent statistical measures that are useful in decomposing non-stationary force data (i.e., DFA) should be considered.

The traditional MVC-task mapping groups’ force output became more regular with neural drive scaling, whereas the novel MVC testing approach revealed consistent irregularity in the force structure across the functional force range. Athreya and colleagues (2012) [21] found similar results in terms of visual feedback, with visual information demonstrating a DFA exponent comparable to the novel MVC group across all force levels, and a DFA exponent in a non-visual feedback condition comparable to that of the higher force levels in the traditional MVC approach. It was proposed that the availability of visual feedback provided an essential override of the system’s intrinsic dynamics, and thus there was more availability of various frequency error corrective processes in order to meet the force output demands. In this vein, it is possible that the novel MVC approach affords individuals the capacity to maintain force level requirements, and thus their DFA profile suggests a greater capacity to utilize more functional processes relevant to corrective control. Another interesting point to consider is the possibility of a traditional MVC approach providing insight into the intrinsic dynamics of the system at particular force levels. That is, individuals who cannot meet the required force may in fact function at a more intrinsically stable force level in an effort to limit their performance error. Such a possibility warrants further experimental inquiry.

There is a growing literature interested in determining distinct force gradation processes relating to isometric force control through both temporal structural analyses and decomposition of their respective frequency contribution. Nagamori et al. (2018) [22], in a simulation study, presented a possible origin of low-frequency oscillation (0–4 Hz.) in the force signal, associated with co-modulation of the collective motor unit pool firing rate known as “common drive”. That is, the organizational firing patterns of individual motor units are centrally controlled on a collective scale as a means to simplify CNS force gradation control. Additionally, Nagamori and colleagues proposed that proprioceptive feedback pathways sufficiently account for the fast frequency contributions (5–12 Hz.) in force output attributed to normal physiological tremor. Their results indicate that modulation and attunement of these neuro-physiological pathways influence the entire frequency spectrum in the force output; however, these inferences have yet to be tested on the full range of individual functional force capacity.

These simulations in conjunction with our findings present two questions for future empirical inquiry. First, given individuals in the traditional MVC-task mapping group demonstrated a higher overall low-frequency contribution (as indexed by DFA); investigation of intrinsically stable force output may provide complementary insight into how a “common drive” pathway may supersede other neuro-physiological corrective processes. Second, given the greater capacity for individuals in the novel MVC-task mapping approach to maintain the required force for the duration of the task, implementing neuro-physiological measures may provide insight into how the organization and interactions of neuro-motor processes at numerous timescales change with biological maturation, aging, and neurological degradation.

There are some limitations to this study that should be noted. A mixed study design was used to control for potential carryover effects associated with learning. However, our sample size was limited (16 subjects per MVC group), and thus a full repeated-measures design, where all subjects perform both MVC conditions, would be useful to eliminate any potential influences of individual differences on our between-group comparisons. Future studies may benefit from examining within-subject changes in force variability characteristics during differential MVC demands to further improve future MVC normalization procedures. Additionally, we limited our group sampling to young healthy adults that did not have significant strength training experience. Future work should examine how the force variability functions (and appropriate MVC calculation procedures) differ when individuals are highly adapted to produce and maintain near-maximal force contractions.

## 5. Conclusions

This study has shown that the temporal mapping of MVC calculation to trial time has a significant impact on behavioral strategy in the control of steady state force gradation. The novel approach to MVC-task mapping in which there is as 1:1 temporal ratio is more appropriate than traditional point peak techniques such as continuous isometric force, and subsequently our results found that the force variability function revealed a sigmoidal trend. Additionally, the MVC approach revealed that individuals presented a more complex force output functioning at multiple timescales through the functional force range.

The differences in estimates of the MVC boundary will depend on the force-time similarity of seeking a MVC determination and whether the averaging techniques are driven by manipulations of force and/or time. Similarly, more precise methods than averaging the force trace could be used to bring the interactive role of fatigue into the force-time relations. Clearly, there are multiple time duration conditions for trials that could be used to estimate the averaged MVC function; however, matching this to the duration of the force variability trials seems to be the most relevant course of action when visual motor precision of force output is of central import.

## Figures and Tables

**Table 1:** Fitted models for RMSE, SD, CE, and DFA as a function of relative force output (%MVC).

**Root Mean Square Error (RMSE)**				
MVC Group:	Fitted Polynomial Function:	R^2^	RMSE	Adjusted R^2^
6s MVC	2nd Order	0.99	0.39	0.99
20s MVC	3rd Order	0.98	0.13	0.98
**Standard Deviation (SD)**				
MVC Group:	Fitted Polynomial Function:	R^2^	RMSE	Adjusted R^2^
6s MVC	2nd Order	0.97	0.39	0.96
20s MVC	3rd Order	0.99	0.07	0.98
**Constant Error (CE)**				
MVC Group:	Fitted Polynomial Function:	R^2^	RMSE	Adjusted R^2^
6s MVC	2nd Order	0.99	0.39	0.99
20s MVC	2nd Order	0.98	0.15	0.98
**Detrended Fluctuation Analysis (DFA)**				
MVC Group:	Fitted Polynomial Function:	R^2^	RMSE	Adjusted R^2^
6s MVC	1st Order	0.84	0.02	0.82
20s MVC	2nd Order	0.52	0.01	0.38

**Table 2:** Mean Differences in RMSE (N).

**Group**	**%MVC**	**5**	**15**	**25**	**35**	**45**	**55**	**65**	**75**	**85**	**95**
6s MVC	5	−	0.263	0.089	−0.367	−0.886 *	−2.671 *	−4.319 *	−5.255 *	−8.173 *	−11.606 *
	15		−	−0.173 *	−0.630 *	−1.149 *	−2.934 *	−4.582 *	−5.518 *	−8.435 *	−11.868 *
	25			−	−0.457 *	−0.976 *	−2.761 *	−4.409 *	−5.345 *	−8.262 *	−11.695 *
	35				−	−0.519	−2.304 *	−3.952 *	−4.888 *	−7.806 *	−11.238 *
	45					−	−1.785	−3.433 *	−4.369 *	−7.287 *	−10.719 *
	55						−	−1.648	−2.584	−5.502 *	−8.934 *
	65							−	−0.936	−3.853 *	−7.286 *
	75								−	−2.917	−6.350 *
	85									−	−3.433
	95										−
	**%MVC**	**5**	**15**	**25**	**35**	**45**	**55**	**65**	**75**	**85**	**95**
20s MVC	5	−	0.330	0.267	0.179	−0.003	−0.251	−0.732	−1.472	−1.538	−2.165
	15		−	−0.063	−0.152	−0.333	−0.581	−1.062	−1.802	−1.868	−2.495
	25			−	−0.089	−0.270	−0.519	−0.999	−1.739	−1.806	−2.432
	35				−	−0.181	−0.430	−0.910	−1.650	−1.717	−2.343
	45					−	−0.248	−0.729	−1.469	−1.535	−2.162
	55						−	−0.480	−1.220	−1.287	−1.913
	65							−	−0.740	−0.807	−1.433
	75								−	−0.067	−0.693
	85									−	−0.626
	95										−

Post-hoc comparisons of Root Mean Squared Error (N) All values represent mean differences in RMSE based on %MVC (column-row), with (*) indicating a significant difference at the 0.05 level.

**Table 3:** Mean Differences in SD (N).

**Group**	**%MVC**	**5**	**15**	**25**	**35**	**45**	**55**	**65**	**75**	**85**	**95**
6s MVC	5	−	0.236	0.105	−0.266	−0.643	−1.112 *	−1.616 *	−1.462 *	−2.693 *	−3.392 *
	15		−	−0.131 *	−0.503 *	−0.879 *	−1.348 *	−1.853 *	−1.698 *	−2.929 *	−3.628 *
	25			−	−0.372 *	−0.748 *	−1.217 *	−1.722 *	−1.567 *	−2.798 *	−3.497 *
	35				−	−0.376	−0.846 *	−1.350 *	−1.196	−2.427 *	−3.125 *
	45					−	−0.469	−0.974 *	−0.819	−2.050 *	−2.749 *
	55						−	−0.504	0.350	−1.581	−2.280 *
	65							−	0.155	−1.076	−1.775
	75								−	−1.231	−1.930 *
	85									−	−0.699
	95										−
	**%MVC**	**5**	**15**	**25**	**35**	**45**	**55**	**65**	**75**	**85**	**95**
20s MVC	5	−	0.292	0.243	0.175	0.047	−0.077	−0.405	−0.855	−1.030	−1.296
	15		−	−0.049	−0.116	−0.245	−0.369	−0.697	−1.147	−1.322	−1.588
	25			−	−0.067	−0.195	−0.320	−0.647	−1.097	−1.273	−1.539
	35				−	−0.128	−0.253	−0.580	−1.030	−1.206	−1.472
	45					−	−0.125	−0.452	−0.902	−1.077	−1.344
	55						−	−0.327	−0.778	−0.953	−1.219
	65							−	−0.450	−0.625	−0.892
	75								−	−0.175	−0.442
	85									−	−0.266
	95										−

Post-hoc comparisons of Standard Deviation (N). All values represent mean differences in SD based on %MVC (column-row), with (*) indicating a significant difference at the 0.05 level.

**Table 4:** Mean Differences in CE (N).

**Group**	**%MVC**	**5**	**15**	**25**	**35**	**45**	**55**	**65**	**75**	**85**	**95**
6s MVC	5	−	0.232	0.347	0.586 *	0.971 *	2.647 *	4.172 *	5.308 *	7.823 *	11.247 *
	15		−	0.114 *	0.353 *	0.738 *	2.415	3.939 *	5.076 *	7.591 *	11.015 *
	25			−	0.239 *	0.624 *	2.301	3.825 *	4.961 *	7.477 *	10.900 *
	35				−	0.385	2.062	3.586 *	4.723 *	7.238 *	10.662 *
	45					−	1.677	3.201 *	4.337 *	6.853 *	10.276 *
	55						−	1.525	2.661 *	5.176 *	8.600 *
	65							−	1.136	3.652	7.075 *
	75								−	2.515	5.939 *
	85									−	3.424
	95										−
	**%MVC**	**5**	**15**	**25**	**35**	**45**	**55**	**65**	**75**	**85**	**95**
20s MVC	5	−	0.278	0.340	0.435 *	0.541	0.793	1.179	1.783	1.741	2.415
	15		−	0.062	0.158	0.263	0.515	0.901	1.505	1.463	2.137
	25			−	0.095	0.201	0.453	0.839	1.443	1.401	2.075
	35				−	0.106	0.358	0.744	1.348	1.306	1.980
	45					−	0.252	0.638	1.242	1.200	1.874
	55						−	0.386	0.990	0.948	1.622
	65							−	0.604	0.562	1.236
	75								−	−0.042	0.632
	85									−	0.674
	95										−

Post-hoc comparisons of Constant Error (N). All values represent mean differences in CE based on %MVC (column-row), with (*) indicating a significant difference at the 0.05 level.

**Table 5:** Mean Differences in DFA (α).

**Group**	**%MVC**	**5**	**15**	**25**	**35**	**45**	**55**	**65**	**75**	**85**	**95**
6s MVC	5	−	0.008	−0.010	−0.010	−0.026	−0.083	−0.074	−0.047	−0.104	−0.116
	15		−	−0.018	−0.018	−0.034	−0.090 *	−0.082	−0.055	−0.112	−0.124
	25			−	0.000	−0.016	−0.072	−0.064	−0.037	−0.094	−0.106
	35				−	−0.016	−0.073 *	−0.064	−0.037	−0.094	−0.106
	45					−	−0.057	−0.048	−0.022	−0.078	−0.090
	55						−	0.009	0.035	−0.022	−0.034
	65							−	0.027	−0.030	−0.042
	75								−	−0.057	−0.069
	85									−	−0.012
	95										−
	**%MVC**	**5**	**15**	**25**	**35**	**45**	**55**	**65**	**75**	**85**	**95**
20s MVC	5	−	−0.004	0.018	0.033	0.015	0.045	0.019	0.031	0.015	0.032
	15		−	0.022	0.037	0.019	0.049	0.023	0.035	0.019	0.036
	25			−	0.015	−0.003	0.027	0.001	0.013	−0.003	0.014
	35				−	−0.018	0.012	−0.014	−0.002	−0.018	−0.001
	45					−	0.030	0.004	0.016	0.000	0.017
	55						−	−0.026	−0.014	−0.030	−0.013
	65							−	0.012	−0.005	0.013
	75								−	−0.016	0.001
	85									−	0.017
	95										−

Post-hoc comparisons of DFA scaling exponent (α). All values represent mean differences in DFA based on %MVC (column-row), with (*) indicating a significant difference at the 0.05 level.

## Data Availability

Data will be made available upon request of the corresponding author.
